# Addressing the persistent inequities in immunization coverage

**DOI:** 10.2471/BLT.19.241620

**Published:** 2019-01-10

**Authors:** Mickey Chopra, Zulfiqar Bhutta, Diana Chang Blanc, Francesco Checchi, Anuradha Gupta, Ephrem T Lemango, Orin S Levine, Dafrossa Lyimo, Robin Nandy, Katherine L O'Brien, Jean-Marie Okwo-Bele, Helen Rees, Jane Soepardi, Rachel Tolhurst, Cesar G Victora

**Affiliations:** a World Bank, 1776 G St NW, Washington, DC, 20006, United States of America (USA).; b Center for Global Child Health, Toronto, Ontario, Canada.; c Department of Immunization, Vaccines and Biologicals, World Health Organization, Geneva, Switzerland.; d Epidemiology and International Health, London School of Hygiene and Tropical Medicine, London, England.; e Gavi, The Vaccine Alliance, Geneva, Switzerland.; f International Institute for Primary Health Care, Ministry of Health, Addis Ababa, Ethiopia.; g The Bill & Melinda Gates Foundation, Seattle, USA.; h Ministry of Health, Community Development, Gender, Elderly and Children, Dodoma, United Republic of Tanzania.; i Health Section, United Nations Children's Fund, New York, USA.; j Chemin de la Capite 6, 1295 Tannay, Switzerland.; k Reproductive Health and HIV Institute, University of the Witwatersrand, Johannesburg, South Africa.; l Kepala Sawit E/23, Cinere 16514, Indonesia.; m Department of International Public Health, Liverpool School of Tropical Medicine, Liverpool, England.; n International Center for Equity in Health, Federal University of Pelotas, Pelotas, Brazil.

A key focus of the health-related sustainable development goal (SDG) 3 is universal health coverage (UHC), including access to safe, effective, quality, and affordable essential medicines and vaccines. However, the challenges to achieving UHC are substantial, especially with increased demands on the health sector and with most budgets being static or shrinking.^1^

Immunization programmes have been successful in reaching children worldwide. For example, 86% of the world’s infants had received three doses of diphtheria-tetanus-pertussis (DTP3) vaccine in 2018.^2^ The experiences from such programmes can contribute to UHC, and as these programmes strive to adapt to new global strategic frameworks, such as Gavi, the Vaccine Alliance’s strategy Gavi 5.0 and the World Health Organization’s (WHO) Immunization Agenda 2030, these efforts can inform the progressive realization of UHC. Immunization programmes that can sustain regular levels of contact between health providers and beneficiaries at the community level have enabled new vaccines to be added to routine immunization schedules and other interventions to be delivered to children and their families. In addition, experiences from both polio campaigns and the child health days strategy show that incorporating additional interventions into campaigns can increase coverage of these interventions as well as of vaccinations.^3^^,^^4^

## Improving immunization coverage

Considering how to expand integration efforts and to better focus immunization on the most disadvantaged, including attention to addressing social determinants of health, will be critical for further progress. The Equity Reference Group for Immunization has conducted analyses based on published and unpublished literature, as well as a series of interviews with experts working at global, national and community levels to highlight several related challenges and opportunities. Here we discuss challenges and opportunities related to data quality, vertical immunization programmes, underserved children and gender.

In 2018, 19.4 million children younger than one year of age did not receive DTP3, and approximately 41% of these children live in countries that are polio-endemic, fragile or affected by conflict.^2^ In addition, a growing share of children live in middle-income countries where vulnerability and social exclusion, particularly among the urban poor, prevents many from receiving vaccination. Children living in remote rural areas, although long identified as a target population for immunization programmes, continue to be underserved. Furthermore, immunization programmes often ignore inequities caused by bias and discrimination in response to the social constructs of ethnicity and gender.

### Data quality

There is growing evidence on the reasons these inequities in immunization exist and how to address them. Acting on this evidence is the challenge to increasing coverage, particularly as it will require redistributing resources, prioritizing those who are often subject to discrimination and operating in challenging contexts. Currently, opportunities that are important considerations for immunization decision-makers and implementers exist.

The first opportunity is the improvement of data quality and use of both traditional surveys and new technologies. Approaches such as linking data sets and use of electronic health information systems can facilitate recording and reporting of real-time data. Simple analyses using existing data can also help us better understand key equity issues within countries. For example, in 2018, WHO released an equity analysis of ten countries that Gavi has identified as the highest priority for childhood immunization.^5^ Using Demographic and Health Surveys (DHS), the report presents disaggregated data on, and associations with, DTP3 coverage by key characteristics of children, mothers and households. This type of information can serve as a basis for more detailed explorations at both national and subnational levels, and as a baseline for future efforts to redress equity gaps. New technologies can provide a better user interface and geospatial information gathering, particularly to improve traditional survey methods and tools. Such advances would facilitate new opportunities that big data and artificial intelligence approaches are bringing to public health.

The second opportunity is innovations such as machine learning and use of satellite imagery, which are already improving estimates of how many children live in different geographic areas, and supporting better visualization of data, which health workers can act upon. Polio eradication programming, for example, has shown how the use of granular data through geographic information systems mapping, coupled with surveillance data, can identify children who are hard to reach by the health-care system. Predictive models informed by data across sectors, such as health, protection, transport and telecommunications, could identify pockets of low coverage even where surveys have not been conducted. However, as quality data are only relevant if used at local levels for planning and budgeting, capacity must be built at national and sub-national levels to better use these data to adapt and expand service delivery strategies. These transformative investments will be critical for both immunization programming and UHC, even as discussions of how best to measure UHC continue.

### Vertical programmes

The vertical nature of immunization programmes is a challenge. This organizational structure has enabled robust vaccination gains, but has been implemented without enough attention to how immunization assets can be used more broadly. Identifying the right mix of interventions to integrate with immunization services, informed by cost–benefit and cost–effectiveness analyses, is critical to ensure that integration does not overburden health workers or negatively impact coverage and quality.^4^ At the global level, additional research is needed to further develop an evidence base around new service delivery models and innovations to simplify vaccine delivery for all children, particularly those living in difficult-to-reach areas. Experts point to the success of strategies that use meticulous microplanning to identify the unreached, engage communities and improve reach through public-private partnerships. Indeed, one of the core axes of UHC is that communities own and drive the design and implementation of services. Immunization programmes are well placed to support this, building on the strengths of the WHO’s Reaching Every District approach, which includes community engagement as a cornerstone. In addition, needle-free vaccine administration and thermo-stable vaccines are promising innovations to enable the health system to simplify and expand delivery to marginalized children. Adoption of novel strategies, such as optimizing delivery strategies and doses per container, reduced dosages and adapted target age ranges within campaigns may reduce disease burden in displaced and intermittently accessible populations. Furthermore, the rollout of human papillomavirus (HPV) vaccination in many countries presents new opportunities for reaching adolescents with other services, such as screening programmes and treatment or other vaccines, and provision of information and life skills. This increased reach can facilitate access for adolescents and can reduce costs and burdens related to delivering interventions separately.

### Underserved children

Developing better approaches for children who may be accessible geographically, but who remain underserved is also a challenge. In some cases, children are underserved by commission, that is, their families deliberately avoid vaccination, while others by omission due to a variety of service delivery and social factors leading to intentional or unintentional exclusion. Incorporating the latest thinking around effective behaviour change approaches into programme and communication strategies may provide new opportunities to reach these children. Reaching these children will also require health systems strengthening, improved quality of care, intersectoral and intragovernmental collaboration, and new emphasis on social justice, non-discrimination, civil society engagement and accountability, among other efforts.^6^

### Gender

A final challenge is to ensure that gender is recognized as a critical, cross-cutting, and influencing factor, and ensuring that gender analyses of immunization are not restricted to comparing coverage outcomes between boys and girls. Studies show that maternal education and maternal age are key determinants of whether a child is immunized. As well, the agency and empowerment of women, and women’s access to quality services can affect the likelihood of childhood immunization.^7^ We must identify and test ways in which immunization programmes can mitigate gender-related barriers without undermining, but rather ideally contributing to, women’s empowerment in different settings. HPV vaccination raises additional gender and equity considerations, particularly as services for adolescents can be quite limited in both availability and quality in many settings.^8^

## Addressing inequities

The strategic importance, effectiveness and cost–effectiveness of focusing on the poorest and hardest-to-reach children has been emphasized before.^9^^,^^10^ Equity in immunization may also contribute to building solidarity within countries for UHC, as everyone, across all socioeconomic levels and from a variety of backgrounds, will benefit from increased herd immunity. However, building solidarity for social and health programmes can be a key challenge in settings where the more advantaged people question why they should pay taxes to ensure services for the less advantaged.^11^ Fortunately, immunization programmes are an example of a public good which, when strengthened and expanded, will benefit those same tax-payers, while also benefitting those who have been previously denied this essential intervention. The financial return on investment in vaccines has been found to be up to 44 times their cost.^12^

We must address inequities in immunization not just for the obvious health, financial and political benefits that come from herd immunity and absence of disease, but because without greater achievement in immunization among children living in urban poor, remote rural or conflict settings, it will be impossible to collectively reach our shared goals for primary health care and UHC.

We have highlighted some of the innovations in the field, as well as the existing assets that immunization programmes can bring. However, using the full potential of immunization programmes to advance UHC will require strategic changes, such as increased efforts to integrate with other services and reaching children never reached by the health system.
